# Self-harm in prison: moving towards contextual understanding

**DOI:** 10.3389/fpsyt.2026.1830793

**Published:** 2026-04-30

**Authors:** Bella Magner-Parsons, Hope Kent, Louise Robinson, Czarina Kirk, Huw Williams

**Affiliations:** 1 Department of Psychology, Faculty of Health and Life Sciences, University of Exeter, Exeter, United Kingdom; 2 Lancashire and South Cumbria NHS Foundation Trust, Preston, United Kingdom; 3 Faculty of Biology, Medicine and Health, University of Manchester, Manchester, United Kingdom; 4 Faculty of Neuropsychology, Lancashire and South Cumbria NHS Foundation Trust, Royal College of Psychiatrists, Preston, United Kingdom

**Keywords:** attitudes, deprivation, distress, forensic mental health, prison, self-harm

## Abstract

**Background:**

Rates of self-harm in prisons in England and Wales are historically high. Despite extensive evidence linking self-harm in custody to adversity exposure, psychiatric morbidity, neurodevelopmental conditions, acquired brain injury (ABI), substance misuse, and treatment in prison (including use of solitary confinement and experiences of victimisation), frontline officers frequently characterise self-harm as “manipulative”. Framing self-harm as uncontextualized manipulation is associated with more hostile, and less therapeutic responses and may contribute to punitive institutional practices.

**Rationale:**

In this perspective, we argue that self-harm in prison is best understood as a multidetermined response to cumulative vulnerability within environments defined by profound power asymmetry. Behaviour perceived as instrumental may represent an attempt to regain safety, communicate urgency, or exert limited control in disempowering and, at times, traumatising conditions. Recognising the multiple drivers of self-harm, including trauma, mental illness, neurodivergence, ABI, and structural deprivation, is essential for compassionate and effective intervention.

**Conclusion:**

We contend that replacing simplistic interpretations, and the language of manipulation with contextual, clinically informed interpretations, alongside structural reform and staff support, is necessary to improve safety in custody and reduce harm to both prisoners and staff.

## Introduction

### A crisis of safety and interpretation

Prisons in England and Wales are facing a safety crisis through high rates of self-harm. Recent official statistics indicate record levels of self-harm across the past several years (1). The most recent official statistics indicate a rate of 878 self-harm incidents per 1, 000 prisoners in the 12-month period to June 2025 (1). Over the same period, the rate of individuals who self-harmed was 160 per 1, 000 prisoners, representing a 3.5% increase from the previous year (2). Compared to an estimated 0.6% of the UK general population reporting self-harm in the previous year (3), the annual prevalence in prisoners has been estimated at 5-6% for men, and 20-24% for women (4). Though, it must be noted general population estimates are likely underestimates due to heterogeneity in measurement techniques, and underreporting of self-harm. Self-harm is defined here as including acts with both suicidal and non-suicidal intent (5). Despite advancements in establishing risk factors such as psychiatric morbidities, substance misuse, and exposure to adversity (6), self-harm remains highly stigmatized and frequently misunderstood (7). Uncertainty regarding the interaction of these risk factors continues to shape inconsistent responses across clinical and custodial settings (6, 8).

In prisons, institutional responses have been described as ineffective and ambiguous (9). There is evidence that responses to self-harm from frontline prison staff are shaped by cynicism developed through years of dealing with repetitive and traumatic self-harm incidences, with very little training or resources (10). In several studies systematically reviewed by Hewson et al. (11), frontline prison staff report that they perceive most self-harm incidents to be ‘manipulative’, where behaviour is interpreted as tactical or instrumental rather than expressive. This perception is associated with more hostile and antipathetic responses to self-harm (11), reflecting wider societal narratives framing self-harm as ‘attention-seeking’ (12).

Parsing out the drivers of these perceptions within prisons, which have under-resourced mental health services and organisational cultures prioritising behavioural control, risk management, and operational efficiency, is essential to improve safety in custody (6, 9, 11). This Perspective argues that reframing self-harm towards a more contextual understanding requires attention to both prisoner vulnerability and the structural conditions shaping staff responses.

### Multiple drivers of self-harm in custody

While ‘interpersonal’ objectives of self-injury, including manipulation are endorsed in prison samples (13), these are suggested to be secondary to ‘intrapersonal’ objectives, such as managing distress, and emotion regulation (14). Moreover, self-harm is rarely performed for a single reason (15). Thus, heuristic attributions of self-harm as only ‘manipulative’ may lead to further misunderstanding of self-harm in custody settings. It is well established in the literature that self-harm in prisoners is an indicator of significant distress; and should be taken seriously. Nearly half of prisoners who die by suicide have a prior history of self-harm (16). In the following sections, we outline the interacting drivers and consider the influence of institutional disempowerment in shaping interpretations of self-harm.

## The distress and disempowerment of prisoners

Those who self-harm in prison often describe self-harm as impulsive acts which are difficult to control, related to intense feelings of anger, hurt, and frustration, which arise because of both life-course factors like histories of abuse, as well as current situational factors (10, 17, 18). This establishes some self-harm not as an instrumental behaviour, but as a marker of acute vulnerability within custodial settings.

### Trauma and psychiatric morbidity

Prison populations have disproportionately high rates of childhood adversity (19, 20), interpersonal violence (21), and are vulnerable to further traumatisation in custody settings (22). Trauma exposure is strongly associated with psychiatric morbidity and self-harm (23). Prisoners experience disproportionately high rates of psychiatric disorders, including post-traumatic stress disorder (PTSD), mood disorders, and substance misuse (24, 25), and psychiatric diagnosis is strongly associated with increased self-harm risk (26). For both men and women in prison, factors such as victimisation across the life course, mood disturbance, and neurodisability have been shown to be predictors of lifetime self-harm (27–29). Embedding an understanding of self-harm that encompasses ‘external explanations’, such as adversity across the life-course, or neurodisability, could promote more compassionate responses from prison staff. Gill et al. (30) have argued that compassion is evoked more strongly when a perceived negative behaviour (e.g., self-harm) from an outgroup (e.g., prisoners) is explained by enduring causal forces, which indicate suffering outside of the individual’s control (30). A better understanding of the complex multimorbidity experienced by prisoners could therefore help to reduce compassion fatigue in prison staff.

A related risk associated with high levels of deprivation and lifetime trauma exposure is acquired brain injury (ABI) (31, 32). Prisoners have very high rates of acquired brain injury (see 26, 33 for reviews), and there is a significant association between brain injury and suicidality and self-harm, particularly when accompanied by emotional distress (34). Clinical perspectives indicate that in forensic secure brain injury units, self-harm is misattributed far less as being solely manipulative. Within clinical settings, training provides a basis for understanding the multidetermined nature of self-harm, where the behaviour may reflect an individual’s difficulty in communicating their distress, or be an expression of frustration they deem as less destructive than interpersonal violence or property damage. Whilst this training is designed for a highly specialised clinical workforce, there may be core principles that could be adapted and applied to training for frontline prison officers, who work with a very complex population but with considerably fewer clinical resources and support.

To our knowledge, there are not any high-quality, public-facing training resources specifically supporting frontline prison staff to understand and respond to self-harm. Some of the core principles (including recognition of multidimensional adversity and aiming to understand the life-course of the person) are captured within broader trauma-informed practice frameworks developed for criminal justice settings (see for example (35)); however, these are less specific to self-harm, and actual practical implementation appears to vary considerably across regions and local areas. There is however a clear need for specific, contextual training which will enable staff to hold awareness for, and understanding of, multiple reasons for self-harm could help to shape individualised responses and support.

### Institutional disempowerment

Individuals with these complex life histories then enter prison environments which are characterised by high operational demand, regimented routines, and limited resources. They are not restorative spaces, and custodial regimes frequently intensify vulnerability. Deprivation of liberty, social contact, and activity opportunities contribute to stress and dysregulation, creating conditions where self-harm may be used in the absence of accessible alternatives (6).

The clearest institutional expression of this intensified disempowerment is in the use of punitive isolation or ‘solitary confinement’. In England and Wales, solitary confinement involves the physical isolation of prisoners in their cells for 22–24 hours per day (36). This can be used as punishment for prisoners who break prison rules; to segregate prisoners for the purpose of ‘maintaining good order or discipline’, or where it is judged as being in the prisoners’ own interests. The use of solitary confinement is widely recognised as dehumanising, torturous, and a violation of human rights (37–39). By design, solitary confinement removes agency, social connection, and control, conditions already eroded by incarceration. Punitive conditions such as solitary confinement are associated with up to 6.9 times higher odds of self-harm, even after controlling for age, race, length of incarceration, and mental illness (40).

This is not an isolated finding; Favril et al. (26) meta-analysis of 35 studies involving over 660, 000 prisoners globally identified solitary confinement, disciplinary infractions, and in-prison victimisation as among the most salient prison-specific risk factors for self-harm (26). Women in prison identify that being put on basic regime (where access to facilities and time out of cell are withdrawn), feeling like they are not being listened to, being treated unfairly, being bullied in prison as important antecedents to self-harm (17, 41). Together, these findings locate self-harm not within individual pathology, but within the architecture of the disempowering prison environment itself. Prisons are, after all, an embodiment of the removal of an individual’s power and autonomy. Incarceration is massively stressful, marginalising, and isolating, and prisoners have some of the poorest mental health outcomes of any group in society (42).

Critically, these harms are not evenly distributed. The impact of solitary confinement is not equal across groups of prisoners; there is evidence of disparities in its use. For example, in the USA Henry (43) found solitary confinement was used disproportionately for men, those with multiple mental health disorders, identifying as multiracial, and identifying as bisexual. For women in prison, deprivation of social contact, including concerns over loss of custody and contact with children is of particular concern. In the UK, women are often held hundreds of miles away from their home communities due to the small size of the women’s estate, (44, 45). Women also disproportionately account for over half of prison self-harm incidents, despite making up only 6% of the general prison population (13, 29, 46). Institutional stressors therefore interact with other intersecting disadvantages to create cumulative adversity, a cascading experience of stress.

Research on the social determinants of health demonstrates that disempowerment, rather than material deprivation alone, is a key driver of morbidity and mortality (47), operating across material, psychosocial, and political dimensions. We draw on this framework here because it explains how institutional structures that disempower people shape distress and morbidity, rather than locating harm solely within individuals. These same mechanisms are central to understanding criminalisation. Disempowerment predisposes individuals to both poor health outcomes and behaviours that become subject to criminal sanction, particularly where health, poverty, and violence intersect (48–51). Within prisons, this disempowerment is cumulative and escalating, intensifying through repeated exposure to deprivation, coercion, and loss of agency. Self-harm should therefore be understood not at an individual level as an attempt to manipulate the system, but as a predictable response to sustained disempowerment within it.

A continued political and public focus on the purpose of prisons as punishment, rather than rehabilitation, compounds disempowerment at both individual and institutional levels. These trends operate within a context of chronic prison overcrowding and a wider safety crisis, characterised by unprecedentedly high death rates, assaults, and incidents of self-harm, alongside dangerously low numbers of front-line officers (52). This environment, particularly over-crowding and under-resourcing, restricts access to healthcare and meaningful activity, and increases reliance on punitive and restrictive practices to maintain order. (53) found punitive disciplinary strategies and overcrowding were associated with depression and hostility among prisoners, with even relatively minor deprivations exerting measurable psychological effects. This evidence underscores the role of the prison environment in elevating self-harm risk (54).

These arguments sit within a broader and growing discourse about the effectiveness of incarceration as a response to harm caused by people who have complex and intersecting needs. Organisations such as the Howard League for Penal Reform have long argued for reduced use of imprisonment, particularly for those with complex mental health needs, and for conditions that prioritise dignity over punishment. The Bradley Report (55) specifically examined the extent to which people with mental health problems and learning disabilities could be diverted toward health and social care at multiple points in the criminal justice system, concluding that many such individuals could be much better rehabilitated in the community, rather than in custodial settings. Academic debate has increasingly questioned whether incarceration, as currently practised, can ever effectively respond to individuals whose pathways into the criminal justice system are rooted in lives of profound intersectional disadvantage, trauma, and unmet need (see 56). Abolitionist perspectives contend that the harms described here are not incidental but structural, intrinsic to the nature of punishment itself (see 57). A fuller engagement with these debates lies beyond the scope of this paper, but the evidence presented here makes clear that custodial settings, as currently constituted, are actively intensifying the vulnerability and distress of some of the most marginalised people in society, rather than responding to it.

## The distress and disempowerment of prison officers

These same structural pressures that shape prisoner distress also shape how self-harm is interpreted and responded to by those tasked with managing it. Prison officers are exposed to self-harm repeatedly, over long periods of time, often being first on the scene to extremely traumatic incidents. Nixon (58) autoethnographic account of being a prison officer describes significant desensitisation to self-harm and suicide, and stigma associated with help-seeking for experiences of trauma. Prison staff engage therefore in sustained emotional labour, balancing containment with care in systems often not equipped (e.g., due to resourcing and staff availability), nor designed (i.e., due to prioritisation of containment and security) to provide adequate care (6, 59).

Expressions of care are also not always institutionally rewarded. Nixon (58) describes how officers who demonstrate empathy or relational engagement may be subject to adverse judgements of professional competence, including being labelled pejoratively as ‘care bears’. Repeated exposure to self-harm and suicide, combined with limited organisational support, contributes to desensitisation, compassion fatigue, and burnout (Smith et al., 2019). Smith et al. (2019) further note that defensive expressions of intolerance towards prisoners’ distress often mirror an intolerance of officers’ own emotional responses, reflecting the cumulative burden of unacknowledged emotional labour (60).

Once again, these pressures operate within a wider context of institutional disempowerment. Prison officers are often young, under-trained for complex mental health crises, and tasked with managing extreme distress in overcrowded, under-resourced environments (61–63). Decision-making autonomy is constrained by rigid regimes, risk-averse organisational cultures, and the constant threat of scrutiny or blame following adverse incidents (58). Prison officers in (41) study report feeling unable to help prisoners with mental health problems; ‘A lot of the problems with people in here are mental health problems, which is something we can’t really do a lot about’ (S4:55, p.279). They also describe feeling desensitised to self-harm, describing some as ‘time-wasters’. Women in this study described these prison officers as being ‘cold in their voice, they’re cold in the way they speak to you, they’re cold as in when you’re crying they just shut the door in your face’ (P14:371, p.280). Within such conditions, emotional distancing and simplified interpretations of behaviour may function as adaptive coping strategies for staff attempting to remain operational.

It is within this context that interpretations of self-harm as ‘manipulative’ emerge and persist. Interpreting behaviour as intentional influence rather than acknowledging distress might reduce uncertainty, contain emotional demand, and reassert a sense of control within otherwise uncontrollable situations. However, these interpretations are not benign. Framing self-harm as manipulative increases reliance on stereotypic attributions and reduces the likelihood of therapeutic engagement, thereby increasing emotional labour and burnout rather than alleviating it (64, 65).

Misattribution has ethical and practical consequences. Interpreting self-harm as purely manipulative can legitimise withholding care, reinforce punitive responses, and further entrench compassion fatigue among staff (6, 11, 64). Yet empirical evidence consistently frames self-harm as a response to distress, trauma, or dysregulation, and in custodial settings it may be understood as a ‘normal’ response to an abnormal environment (6, 64, 66). Failure to respond to underlying distress and instead to adopt antipathic responses increases the likelihood that self-harm will be repeated with escalating severity illustrating punitive responses to self-harm are harmful (67).

## Reforming dehumanising systems to challenge misattribution

Challenging the widespread misattribution and stigmatisation of self-harm as manipulation requires a systemic response that attends to both prisoner vulnerability and staff working conditions. Prison officers are routinely expected to manage extreme distress in the absence of adequate training, therapeutic support, or well-resourced prison mental health services. At the same time, prison populations have high rates of complex vulnerability, including acquired brain injury, trauma exposure, and mental ill-health, all of which are associated with emotional dysregulation and elevated risk of self-harm (see 33, 34 for reviews). These vulnerabilities are socially patterned, reflecting longstanding and ongoing inequalities in access to early intervention, trauma-informed care, and community health support, particularly for individuals facing racialised, gendered, and disability-related disadvantage (68). Thus, any approaches aiming to improve conditions must account for systemic disadvantage and inequalities.

Mental health services in prisons are chronically underfunded, mirroring broader constraints in the mental healthcare system, including provision of hospital beds and community-based support (69). Declining mental health service availability and growing prison populations have been hypothesised to be inversely associated (see Penrose hypothesis; (70, 71)). Under this perspective, prisons operate as surrogate sites of containment for those with complex psychiatric needs where mental healthcare provisions are too restricted in availability, and are simultaneously ill-equipped to offer the conditions of treatment needed for recovery. This is reflected in empirical evidence supporting the association between declining availability of hospital beds in the UK, and a corresponding increase in prison populations (72, 73). The House of Commons Justice Committee (74) reports that mental health resources are seriously inadequate, with only around 10% of prisoners in the UK receiving interventions for mental health at any one time whilst an estimated 70% are in need at any one time. In addition, these mental health services may not be well adapted to meet the needs of prisoners with intersectional disadvantage; those with neurodisability, for example, or those experiencing gendered poly-victimisation. These problems are persistent and entrenched, requiring rethinking on solutions, particularly at a broader systemic level, to address them. This is reflected in the statement that, in the UK: “prison has become a ‘catch-all’ social and mental healthcare service, and a breeding ground for poor mental health” (75).

Meaningful reform of self-harm responses in prisons must begin with the conditions that generate distress and disempowerment. As demonstrated above, self-harm in custody is not an aberrant behaviour but can be a predictable response to environments characterised by deprivation, isolation, and loss of control. Chronic overcrowding, the routine use of solitary confinement, and a sustained shift toward punitive prison regimes produce conditions of misery and desperation that are likely to increase both the incidence and severity of self-harm. Without addressing these systemic drivers, individual interventions will remain limited in their effectiveness.

Within this context, the Assessment, Care in Custody and Teamwork (ACCT) process, a safety process aiming to reduce risk of self-harm and suicide in UK prisons operates under significant constraint. Although ACCT enables multidisciplinary assessment, implementation often prioritises containment over engagement with underlying distress. Staff report limited time for meaningful interactions with prisoners, while prisoners report sleep disruption attributed to nighttime observations, and a lack of clarity for why they are under intense observations. While environmental safeguards are necessary, ACCT and related processes must function as supportive interventions rather than procedural safeguards alone. This requires adequate staffing, access to mental health services, and organisational cultures that prioritise relational engagement. Repeated self-harm should be interpreted as evidence of unmet need, rather than manipulation or non-compliance (76, 77).

Other approaches which are less formal and less clinically focussed also show promise and should be invested in further, such as the Samaritans Listeners scheme (78), and other peer and social support programmes (see Walton et al. (79) for comprehensive overview)). An important caveat is that such schemes should not be relied upon to substitute for care that remains the responsibility of the state. However, when adequately funded and with appropriate support structures for peer mentors themselves, these programmes can provide meaningful support for prisoners who may distrust formal services and who stand to benefit from connecting with others who have been through similar experiences.

Staff training and support are important, but secondary to structural reform. Trauma-informed approaches may reduce heuristic instrumental interpretations (64), yet sustained empathetic engagement is unrealistic in chronically overcrowded and crisis-driven environments. Effective reform must therefore combine staff education with reductions in isolation, investment in mental health provisions including non-clinical support initiatives, and explicit rejection of punitive responses that exacerbate harm. Our key recommendations for improving responses to self-harm in custody are in **Table 1**.

**Table 1:** Key recommendations and brief rationale for improving responses to self-harm in custody.

Domain	Recommendation	Rationale
Institutional Reform	Reduce overcrowding and limit use and length of solitary confinement.	Addresses some key structural drivers of distress.
Increase provision of non-clinical support	Continue to increase investment in third-sector initiatives such as the Samaritans Listeners scheme (78), and peer support programmes (see Walton et al. (79) for comprehensive overview)).	Addresses distress socially, potentially reducing burden on formal clinical services, and provides an alternative for support where clinical approaches may not be preferred.
Staff Education	Mandatory training covering the complexity of self-harm. Modules should include training on trauma exposure, neurodivergence, and ABI and link how these experiences may increase vulnerability to self-harm.	Enhances contextual understanding and reduces moral attribution.
Involvement of Experts by Experience	Training should be co-produced with experts by experience, though this must be fully resourced to manage the emotional effects of coproduction and ensure ethical and sustainable engagement (see Burnham et al., 2025 (80) for review).	Lived experience involvement addresses epistemic and practical limitations of professionally led approaches in custodial settings.
Staff Support	Provide supervision and psychological support for staff, particularly frontline officers.	Mitigates burnout and compassion fatigue.
Mental Health Investment	Increase access to timely psychological, psychiatric, and social intervention.	Provides access to diagnosis and treatment while targeting psychiatric morbidity and provides support for regulating distress, including involvement of non-clinical approaches as an additional source of support.
Neurodisability Screening	Routine screening for neurodisability, including ABI using tools such as the ‘Do-It’ Profiler (81). Though, cost efficacy of screening requires evaluation and ethical oversight to ensure individuals provide consent to screening.	Identifies underlying vulnerability to allow for individualised understanding, and identifies burden of need.
Policy Language	Guidance on self-harm in custody must be clear, specific, and easy to follow. Compassionate, judgement-free language should be used when discussing any aspect of self-harm.	Promotes clinically grounded interpretation and establishes clear guidance on how to talk about self-harm.
ACCT Enhancement	Prioritise relational engagement alongside observation.	Moves beyond containment-only approaches.

ABI, Acquired Brain Injury.

## Conclusion

Self-harm in prison cannot be reduced to a single motive or moral category such as ‘manipulation’. Self-harm in prisons reflects the interaction of disproportionate lifetime trauma exposure, psychiatric morbidity, and cumulative institutional deprivation. Some acts may be for instrumental purposes, particularly where prisoners seek to change their immediate circumstances (e.g., spend time in the hospital wing). However, apparent instrumentality does not negate profound distress. In environments characterized by restricted autonomy and pronounced power asymmetry, attempts to alter conditions may represent understandable responses to perceived lack of control, and sometimes brutal conditions.

Evidence suggests that punitive or dismissive reactions may escalate harm, increasing risk for prisoners and compounding trauma exposure for staff. Replacing the language of manipulation with contextual, clinically informed interpretation aligns practice with empirical evidence. Such reframing may reduce stigma, improve relational therapeutic engagement, and mitigate punitive responses. However, reframing must be accompanied by structural reform addressing overcrowding, isolation as punishment, and inadequate mental health provision. The distress and disempowerment described in this paper is intrinsic to justice responses rooted in punishment rather than rehabilitation, and to prison overcrowding as a result of under-use of diversion. The individuals described here could be better served by community-based health and social care responses, including relational peer-support initiatives, and a broader reorientation of our justice system is necessary not only to better care for vulnerable people within it, but also to create safer communities with less reoffending.

Improving safety within custodial settings requires recognition that self-harm is most often a marker of vulnerability within systems that compound disadvantage. Supporting staff to understand the multiple drivers of self-harm, alongside advocating structural reform, is an essential step towards recognising the multidetermined nature of self-harm and resisting reductive narratives of manipulation. A clinically grounded approach requires moving beyond binary interpretations of authenticity versus strategy. Psychiatric practice routinely accommodates complexity; justice systems must do the same if they are to improve safety in custody.

## Data Availability

The original contributions presented in the study are included in the article/supplementary material. Further inquiries can be directed to the corresponding author.
